# Fellowships, Grants, & Awards

**Published:** 2007-04

## Methodology and Measurement in the Behavioral and Social Sciences (R01)

The behavioral and social sciences offer insights into the comprehensive understanding of human health, including disease etiology and treatment, and the promotion of health and well-being. To encourage the investigation of the impact of social and behavioral factors on health and disease, the participating institutes and centers invite qualified researchers to submit research grant applications on methodology and measurement in the behavioral and social sciences. Methodology and measurement encompass research design, data collection techniques, measurement, and data analysis techniques. The goal is to encourage research that will improve the quality and scientific power of data collected in the behavioral and social sciences, relevant to the missions of the NIH Institutes and Centers (ICs). Research that addresses methodology and measurement issues in diverse populations, issues in studying sensitive behaviors, issues of ethics in research, issues related to confidential data and the protection of research subjects, and issues in developing interdisciplinary, multimethod, and multilevel approaches to behavioral and social science research are particularly encouraged, as are approaches that integrate behavioral and social science research with biomedical, physical, or computational science research or engineering. Applicants are encouraged to contact the Program Contact for Scientific/Research issues (see “Agency Contacts”) of the IC that most closely matches their research focus to determine the IC’s interest in the research topic.

This Funding Opportunity Announcement (FOA) encourages applications addressing four general areas of methodology and measurement research in the social and behavioral sciences. These areas include research design, data collection techniques, measurement, and data analysis. Within the broad spectrum of research defined by these areas, applicants are encouraged (but are not required) to consider studies that address one or more of the following key issues: 1) methodology and measurement issues in developing innovative interdisciplinary, multimethod, and multilevel research designs for use in behavioral and social science research, with emphasis on developing new technologies and addressing the analytical complexities associated with the integration of behavioral, social, and biological data; 2) methodology and measurement issues in research relating to diverse populations, e.g., populations that are distinctive by virtue of age, gender, sexual orientation, ethnicity, culture, including culture-specific medical systems, socioeconomic status, literacy, language, or disability; 3) methodology and measurement issues in studying how dramatic changes in economic, social, environmental, physical, or political context affect human health and well-being, including developing new methods if older ones are no longer valid in the face of significant changes in populations and societies over the last several decades; 4) methodology and measurement issues in studying potentially sensitive behaviors, such as sexual behavior and abortion, and covert or illegal behaviors such as drug use, abuse, and violence; 5) methodology and measurement issues concerning ethics in research, with emphasis on the topics of informed consent, assessment of risk and benefit, and selection and retention of subjects, and ensuring subjects’ confidentiality.

Multidisciplinary and interdisciplinary approaches are strongly encouraged. Potential applicants are urged to explore the ideas and methods developed in social science and behavioral fields other than their own and to consider the development and integration of behavioral and social science measures with those of the biomedical, physical, or computational sciences or engineering. Consulting relevant literature and collaborating with colleagues from other disciplines may provide important opportunities for cross-fertilization in developing improved methodology and measurement.

### Research Design

Research design determines how well a research plan can accomplish stated purposes and test hypotheses. Research design encompasses many decisions including the sampling plan; selection of appropriate study designs, methods, procedures and measures; and assuring confidence in the study’s internal and external validity. An innovative sample design or sampling frame can be the centerpiece of a research design. Examples of topics within research design include the following: 1) conceptual design strategies appropriate for the study of culture in the context of health; culture may include ethnic group, professional affiliation (e.g., culture of biomedicine), culturally specific medical system, or interaction of multiple subcultures within a health-related context; 2) designs to improve causal inference from nonexperimental and quasi-experimental research and natural experiments; 3) research to improve the efficacy of multilevel designs; 4) methods for improving the design and evaluation of community-based research and intervention trials (e.g., health promotion/disease prevention programs); 5) conceptual, methodological, and ethical issues in designing studies that use different sources of information; e.g., studies comparing self- and third-party reports (e.g., from participants’ family or friends); 6) designs to improve and compare various approaches to economic analysis, including cost analysis, cost-effectiveness, cost–benefit, and conjoint analysis for improving decision-making in health policy and health care systems; 7) designs to improve the inclusion of underrepresented groups in research, e.g., women; children; the elderly; ethnic and racial minorities; sexual minority groups; geographically isolated groups; mentally or physically impaired or disabled populations; immigrants and refugees; and language minority populations; 8) designs that allow for the meaningful integration of biological, behavioral, and social science data (e.g., to study interactions among genetics/genomics, behavior, and the social environment); 9) ethical considerations in research design including issues of informed consent, assessment of risk and benefit, confidentiality, and selection and retention of subjects; 10) methods for archiving and disseminating complex data sets, especially longitudinal data sets, data sets including social network data or geographic identifiers so the identities of study participants are protected and so the data sets can be used by other investigators; 11) alternatives to randomized, controlled trials, including studies to investigate the feasibility, validity, and benefit of incorporating subject (e.g., clients, patients, families, clinicians) preference in intervention design and outcomes assessment; issues regarding blinding; and design of placebo and other control groups; 12) methods to improve the design of clinical research for enhancing quality of patient care (e.g., studies linking patient-reported outcomes data with medical records and/or billing information to improve patient–provider communication, patient decision making, or clinical decision making); 13) designs that allow assessment of the active components of multilevel interventions; 14) methodology to ensure the integrity and consistency of data obtained in multisite studies.

### Measurement Issues

Developing and validating research instruments and questions are vitally important for collecting reliable information, and have obvious impact on data validity and reliability. For example, health care practitioners must collect reliable reports of symptoms from their patients to accurately diagnose disease. In addition, data collection instruments and questions developed for a particular age, gender, or cultural group may not be valid for other groups. For example, a dietary history questionnaire developed for Americans of European descent may not contain the foods commonly eaten by Americans of African, Asian, or Hispanic descent. Specific consideration of the processes underlying potential bias in self-report data collection remains a measurement issue: perceptual, cognitive, cultural, demographic, motivational, and affective influences on self-report data. Finally, continued improvement and innovation in developing and validating data collection instruments are important for all types of research settings, ranging from the clinical interview and observational study to the survey. Examples of measurement issues include: 1) development and refinement of measures, instruments, or surveys used in behavioral and social science research that fill a gap in research needs, with an eye toward developing and validating core sets of items to reduce redundancy across research projects; 2) instrument design, calibration, and refinement; instrument design issues in studying age, gender, and culture, including methods of studying culture and self-identification of race/ethnicity, as well as the psychometric properties underlying data collection instruments; 3) measurement issues in using technology such as computer-assisted data collection, web-based technology and personal digital assistants; 4) direct and indirect measurement of attitudes, values, self-esteem, and other psychological variables; this includes examination of economic values, including willingness-to-pay, as a means of evaluating benefits; 5) development of instruments that assess degree of change, and rate and variable direction of change; 6) development of instruments or technologies that measure behavior objectively or reduce self-report burden; 7) development of objective measures of components of the built environment that ffect health and of measures for assessing interactions between people and the built environment; 8) methodology to objectively measure the cultural and social environment, including aspects of the community environment that influence health; 9) methodology for validating geographic information system (GIS) data obtained from different sources; 10) methodology to measure fidelity to behavioral interventions, including the development or adaptation of behavioral and interpersonal measures to better assess the fidelity of community-based and multisite behavioral interventions; 11) development of measures of self-management of chronic illness and disability and of family management of chronic illness and disability among children; 12) development of behavioral and social science measures that can be used for efficient data collection in clinical practice–based research networks; 13) methodology to capture the behaviors of health care providers in clinical settings; 14) methods that incorporate relevant biological, physical, or computational measures into behavioral and social science research; 15) development of measures for evaluating quality of life near the end of life in a variety of populations, and refinement of existing measures through longitudinal studies and across various disease states.

### Data Collection Techniques

Data collection techniques are the tools and procedures scientists use for implementing research designs and obtaining measurements. Methods for collecting research data have an important impact on data validity and reliability. For example, studies have suggested that use of self-administered instruments can facilitate the reporting of sensitive or illegal behaviors. Innovative methodologies can also open the door to the collection of new or more complex types of data by behavioral scientists. For example, recent developments in computer-assisted interviewing have permitted more complex question sequences in survey research and the development of hand-held “beepers” programmed for data entry have permitted the collection of time-specific data on activities such as cigarette smoking. In addition, implicit measures have allowed researchers to examine processes of which people themselves have been unaware. Continued improvement and innovation in data collection methods are important for all types of research settings, including clinical interviews, observational studies, participatory action research, and surveys. In addition, more research is needed to understand how various methods work in diverse populations, and how they can be modified to address the specific needs of populations. Potential topics within data collection techniques include, but are not limited to the following: 1) methods to improve data collection in surveys and epidemiologic self-report studies, ethnographic and other qualitative studies, participatory action research methods, and multimethod studies; this may include new approaches to instrument design and manipulation of method and mode of data collection, length, setting, and interpersonal factors in data collection exchanges; 2) methods to develop innovative gold standards to assess and improve the accuracy of self-reports, in the absence of health record checks (a possible consequence of the Privacy Rule of the Health Insurance Portability and Accountability Act); 3) new methods for qualitative research; methods for validating narrative or text-based analyses; techniques for validating and replicating findings from qualitative research, including collection strategies, development of coding protocols, and techniques that facilitate the integration and validation of qualitative and quantitative measurement; 4) methods to reduce sampling, survey, and item nonresponse bias in research studies, including techniques to improve the coverage of relevant populations in household surveys, to increase the voluntary participation of eligible subjects, to reduce attrition in longitudinal studies and clinical trials, and to improve response rates on sensitive items; 5) techniques for collecting contextual data (e.g., neighborhood composition, peer group characteristics, geographic and environmental information) and for operationalizing the boundaries of particular social, economic, physical, and cultural contexts; 6) improved technologies for data collection, including automated collection and reporting technologies, such as computer-based data collection and computer adaptive methodology, and research on how the method/mode used to collect data affects the quality of the data in a variety of populations and substantive areas; 7) data collection techniques that address the needs of special populations (e.g., physically or mentally disabled, nonliterate populations, non-English speaking populations, the homeless, the incarcerated, children, the elderly, critically ill patients) and that address how these methods affect data quality and completeness across diverse populations; 8) issues surrounding the collection of self-report data from different settings (e.g., alone, in groups), different collection methods (e.g., oral, written), from different parties (e.g., participants, third parties), and the use of implicit and explicit measures; 9) development of better data collection techniques for studies that involve populations which are small or difficult to access, such as the terminally ill.

### Analytic Methods

Analytic methods encompass the concepts and techniques used in analyzing data and interpreting and reporting results, to improve estimation, hypothesis testing, and causal modeling based on scientific data. Challenges include developing techniques that distinguish underlying regularities from the noise created by variability and imprecise measurement; developing causal inferences from nonexperimental data; improving both the internal validity and external validity (generalizability) of measures and studies; and developing appropriate analytic techniques for use with new kinds of data and new approaches to behavioral and social science research, and meaningful integration of behavioral and social science data with those obtained from the biological, physical, computational sciences, or engineering. Examples of topics within analytic methods include: 1) research to improve the analysis of longitudinal data, in particular, the analysis of correlated data, the modeling of different sources of error, and techniques for dealing with missing data at various levels of aggregation; 2) methods for improving the analysis of multicultural; community-based multilevel intervention trials (e.g., health promotion/disease prevention programs); 3) methodological research to improve the analysis of complex survey data, including the statistical modeling of nonresponse and other survey errors; 4) analytic issues in innovative techniques for improving causal inference from nonexperimental research; 5) analytic methods for integrating evidence from qualitative and quantitative research, such as research examining the complex relationships among multiple sources of information on a single construct (e.g., self- and third-party reports, clinical examinations and testing, laboratory tests, and other record sources); 6) analytic methods that appropriately model social structures, social processes, and spatial relationships such as social networks, social influence, diffusion, and contextual effects; 7) statistical procedures for accurately estimating multilevel models; 8) development of novel mathematical and computational techniques for analyzing and modeling behavioral and social processes; 9) methods for improving the analysis of nonindependent data, such as data examining processes in interactions between couples, families, or other groups; 10) developing sophisticated analytic methods that take into account complex probability designs used in epidemiological research; 11) analytical methods to incorporate geographic and built environment measures into behavioral and social science research; 12) expanding current psychometric methodologies to handle the types of data collected in behavioral and social science research, e.g., multidimensional data, shorter scales, nonnormal score distributions, mixed response format, and complex survey structure; 13) methods for the detection and analysis of nonlinear or discontinuous changes in response to treatment.

### Relevant Research Links

Potential applicants specifically concerned with research regarding the inclusion of language minorities should see the 2001 report on the conference “Diverse Voices—The Inclusion of Language-Minority Populations in National Studies: Challenges and Opportunities,” sponsored by National Institute on Aging, the National Institute of Child Health and Human Development, and the National Center on Minority Health and Health Disparities (see http://www.nichd.nih.gov/publications/pubs/diverse_voices.htm).

In June 2000 the Office of Behavioral and Social Sciences Research held a conference “Toward Higher Levels of Analysis: Progress and Promise in Research on Social and Cultural Dimensions of Health.” After the conference, a panel of scientists developed an ambitious research agenda on the social and cultural dimensions of health. A program announcement based on the panel’s recommendations for substantive research has been issued by OBSSR (see http://grants.nih.gov/grants/guide/pa-files/PA-05-029.html). However, the research agenda also included detailed recommendations relating to needed methodological research related to the social and cultural dimensions of health. Potential applicants are encouraged to consult this report (see http://obssr.od.nih.gov/Conf_Wkshp/higherlevel/conference.html).

In September 2001, NIH sponsored an International Conference entitled: Stigma and Global Health: Developing a Research Agenda. The recommendations included 1) encouragement of research intended to develop methodological, evaluative, and analytic tools for studying stigma and its consequences with respect to health, and 2) development, evaluation, and optimization of interventions to prevent or mitigate the negative effects of stigma and discrimination on health. In both areas the social and cultural dimensions of stigma and its manifestations should be included. Applicants are encouraged to refer to the stigma conference website: http://www.stigmaconference.nih.gov for further resources and information.

In addition, the following reports may be useful as general references on behavioral and social sciences research as it relates to health:

New Horizons in Health: An Integrative Approach (2001) (http://www.nap.edu/catalog/10002.html).

Health and Behavior: The Interplay of Biological, Behavioral, and Societal Influences (2001) (http://books.nap.edu/catalog/9838.html).

From Neurons to Neighborhoods: The Science of Early Childhood Development (2000) (http://books.nap.edu/catalog/9824.html).

Bridging Disciplines in the Brain, Behavioral, and Clinical Sciences (2000) (http://books.nap.edu/catalog/9942.html).

Expanding the Boundaries of Health and Social Science: Case Studies in Interdisciplinary Innovation (2003). Frank Kessel, Patricia Rosenfield and Norman Anderson, Editors. New York:Oxford University Press.

Rebuilding the Unity of Health and the Environment (2001) (http://www.nap.edu/books/030907259X/html/).

Cells and Surveys: Should Biological Measures be Included in Social Science Research? (2001) (http://www.nap.edu/books/0309071992/html/).

Qualitative Methods in Health Research: Opportunities and Considerations in Application and Review (2001) (http://obssr.od.nih.gov/Documents/Publications/Qualitative.PDF).

Applicants may also wish to consult the following report on the protection of human subjects in behavioral and social sciences research: Protecting Participants and Facilitating Social and Behavioral Sciences Research (2003) (http://newton.nap.edu/catalog/10638.html).

This FOA will use the NIH Research Project Grant (R01) award mechanism. The applicant will be solely responsible for planning, directing, and executing the proposed project. This FOA uses “Just-in-Time” information concepts. It also uses the modular as well as the nonmodular budget formats (see http://grants.nih.gov/grants/funding/modular/modular.htm).

Specifically, if you are a U.S. organization and are submitting an application with direct costs in each year of $250,000 or less (excluding consortium Facilities and Administrative [F&A] costs), use the PHS398 Modular Budget component provided in the SF424 (R&R) Application Package and SF424 (R&R) Application Guide (see specifically Section 5.4, “Modular Budget Component,” of the Application Guide).

U.S. applicants requesting more than $250,000 in annual direct costs and all foreign applicants must complete and submit budget requests using the Research & Related Budget component found in the application package for this FOA. See NOT-OD-06-096 23 August 2006. Applicants must download the SF424 (R&R) application forms and the SF424 (R&R) Application Guide for this FOA through Grants.gov/Apply.

Note: Only the forms package directly attached to a specific FOA can be used. You will not be able to use any other SF424 (R&R) forms (e.g., sample forms, forms from another FOA), although some of the “Attachment” files may be useable for more than one FOA.

For further assistance, contact GrantsInfo: 301-435-0714, (telecommunications for the hearing impaired: TTY 301-451-0088), or by e-mail: GrantsInfo@nih.gov.

The application submission dates for this PA are available at http://grants.nih.gov/grants/funding/sub-missionschedule.htm. The complete version of this PA is available at http://grants.nih.gov/grants/guide/pa-files/PA-07-060.html.

Contacts: The complete list of agency contacts is available at http://grants.nih.gov/grants/guide/pa-files/PA-07-060.html.

Reference: PA-07-060.

